# Prognostic value of high-resolution magnetic resonance imaging in evaluating carotid atherosclerotic plaque in patients with ischemic stroke

**DOI:** 10.1097/MD.0000000000008515

**Published:** 2017-11-10

**Authors:** Jin-Er Shu, Ming-Liang Ying, Xiao-Rong Chen, Jian-Jun Hua, Jie-Ting Fu, Xiu-Mei Xia, Yong-Hao Pan, Yang Jiang

**Affiliations:** Department of Radiology, Jinhua Hospital of Zhejiang University, Jinhua, P.R. China.

**Keywords:** carotid atherosclerotic plaque, high-resolution magnetic resonance imaging, ischemic stroke, prognosis

## Abstract

**Background:**

Ischemic stroke (IS) is a devastating occurrence affecting millions worldwide. This study aimed to evaluate the prognostic value of high-resolution magnetic resonance imaging (HRMRI) in assessing carotid atherosclerotic plaque in IS patients.

**Methods:**

Between January 2013 and March 2015, 338 IS patients were recruited for the investigative purposes of the study. All participants of the study underwent an HRMRI inspection procedure after being admitted into the hospital. During this study, we systematically analyzed and measured various types of fibrous caps, lipid compositions, and plaque lipid ratios. Univariate and multivariate logistic regression analyses were performed for predicting prognosis of IS patients. A receiver-operating characteristic (ROC) curve was employed to determine the accuracy of the IS prognosis.

**Results:**

The percentage of type I fibrous caps exhibited significant decrease, while the percentage of type III fibrous caps, lipid compositions, and lipid ratios all displayed increase. The results of the univariate analysis indicated that age, hypertension, hyperlipidemia, treatment regimens, fibrous cap type, plaque type, lipid composition, and lipid ratio shared a correlation in regards to the poor prognosis of IS patients. Multivariate logistic regression analysis demonstrated that the prognosis of IS patients was not necessarily dependent on fibrous cap type, plaque type, or age. ROC curves revealed that the HRMRI possessed a strong predicative ability in relation to the identification of the prognosis of IS patients through factors such as type of plaque and fibrous caps determination.

**Conclusion:**

Our study conclusively intimated the promise of HRMRI as an evaluative tool for the determination of carotid atherosclerotic plaques in patients with IS.

## 1 Introduction

Stroke remains a major cause of death and decrepitude worldwide, frequently rendering its victims powerless from the damage that it causes to the human body as well as motor functioning capabilities. Stroke and stroke-related ailments at present are large contributors to the rising costs of health care despite intensive efforts and significant developments in the field of neuroscience.^[1]^ Approximately 87% all occurring strokes are of the ischemic stroke (IS) kind around the world.^[2]^ IS currently ranks the 4th among commonly occurring fatal diseases. This consequently leads to serious economic implications as well as emotional burdens to the patients and their families.^[3]^ The clinical causes of IS are both complicated and not fully understood, while the most common carotid atherosclerosis has been highlighted as the chief cause of IS.^[4]^ Currently, approximately 20% of IS cases are caused by carotid atherosclerosis. Carotid atherosclerosis is a chronic inflammatory process that is often accompanied by cholesterol deposition and progressive thickening of the arterial wall, which distresses the blood supply to the brain and reduces the cerebral blood flow as well as collateral circulation. That eventually leads to arterial thrombosis, while significantly increases the susceptibility to IS.^[5]^ Thus, a preventive strategy for patients suffering from IS is required for the early detection, diagnosis, and treatment of IS.

With the progressive development of modern imaging techniques, various imaging modalities are gradually being employed as diagnostic tools for the treatment of IS, including angiography, color-coded duplex sonography, magnetic resonance angiography, and computed tomography (CT) angiography.^[6–8]^ However, there are many drawbacks that accompany the aforementioned imaging techniques in regards to the diagnosis of IS, including the inability to display lesions on the vessel wall structures.^[9]^ Recent studies have indicated that the use of high-resolution magnetic resonance imaging (HRMRI) technology provides more clinical valuable information in relation to obtaining information concerning vessel wall structures and plaque compositions at lesion sites.^[10,11]^ HRMRI not only detects the formation of atherosclerosis accurately, but also provides information on atherosclerotic plaques including the core of necrotic plaque, the location of intracranial hemorrhage, and the onset of inflammation and neovascularization, which in turn aids clinicians’ predictive powers in relation to the stability of vascular damages and the occurrence of vascular diseases.^[12]^ Furthermore, HRMRI is also capable of identifying mechanisms of intracranial atherosclerotic IS through detecting luminal plaques.^[13]^ Relatively few studies have explored the associations of HRMRI with the carotid atherosclerotic plaques in IS patients. Therefore, our study aimed to systematically evaluate the diagnostic value of HRMRI in estimating the value of carotid atherosclerotic plaques in IS patients.

## 2 Materials and methods

### 2.1 Ethic statement

The present study was performed in accordance with the preset guidelines established by the Medicine Ethics Review Committee of the Jinhua Hospital of Zhejiang University. All patients participating in the study signed written informed consent documentation, prior to the initiation of the study.

### 2.2 Subjects

A total of 338 IS patients were recruited from the Department of Neurology at the Jinhua Hospital of Zhejiang University between January 2013 and March 2015. The recruited patients consisted of 186 males and 152 females aging 45 to 83 years old (mean age 64.75 ± 8.14 years). The inclusion criteria of the study were: all patients had to undergo head CT or magnetic resonance imaging (MRI) examinations 3 days prior to hospital admittance, and the test results were in line with the World Health Organization diagnostic criteria for IS^[14]^; CT or MRI imaging results obtained confirmed significant infarction in the arterial blood supply regions of the brain and the anterior cerebral artery. Patients displayed neurological deficits in the blood supply regions of the carotid artery and the symptoms continued 24 hours or more. The exclusion criteria were: IS that was caused by fibrillation, infectious endocarditis, vasculitis, or arterial dissection; patients with malignant tumors, diseases of circulatory system, or other severe infections; patients with severe lung, heart, liver, or kidney diseases; patients that were at the time of testing in the acute phase of IS and were currently receiving thrombolysis or endovascular treatment procedures; and patients who recently underwent surgery or had a history of trauma. All patients in the study underwent HRMRI examinations after being admitted to the hospital.

### 2.3 HRMRI examination

HRMRI was performed using a 3.0 T MRI scanner (Achieva TX, Philips Healthcare, Best, The Netherlands) with a carotid dedicated 4-channel phased-array surface coil. The system was positioned parallel to the line that connected the anteroposterior edge of foramen magnum. Initial scans were conducted using standard carotid 2D high-resolution MRI sequences in order to obtain the initial position: axial 3D TOF and 2D-BB-MRI placed at the center of the bilateral carotid artery bifurcation and the 2 cm region around the center was subsequently scanned. The plaque composition (lipid-rich core, intraplaque hemorrhage, and calcification) and the surface condition of the fibrous cap of the plaque (complete or broken) in the common carotid artery, carotid bifurcation and internal carotid artery as references, were observed and used to assess the stability of the plaque. The 3D TOF was performed based on the following parameters: using multiple overlapping thin slab acquisition; echo time (TE), 3.4 ms; repetition time (TR), 29 ms; field of view (FOV), 240 mm × 240 mm; matrix, 256 × 256; seam thickness, 1.2 mm; interlayer spacing, 0.6 mm; and nonrange scanning. Next, the patients underwent a 3D-BB-MRI scan, T1-weighted (T1WI): T1WI: TE, 15.8 ms; TR, 567 ms; FOV, 100 mm × l00 mm; seam thickness, 2 mm; matrix, 256 × 256; interlayer spacing, 2.5 mm, T2-weighted (T2WI): T2WI: TE, 49 ms; TR, 2883 ms; FOV, 100 mm × 100 mm; seam thickness, 2 mm; matrix, 256 × 256; interlayer spacing, 2.5 mm; and proton density-weighted image. 3DTOF images of the carotid artery bifurcation were regarded as the scan locations in the unilateral carotid lumen. The FOV orientation was parallel to the long axis of the carotid artery and covered a minimum of an area of 2 cm around the carotid bifurcation, thus producing 3D-BB-MRI oblique sagittal images on one side of the carotid artery. The standard axial rebuilding of images was completed after acquiring the original images using 3D-BB-MRI. Following the matching process of 2D-BB-MRI and 3D-BB-MRI images, measurement and comparisons were completed based on plaque morphology as well as the degree of stenosis on a relative matching level. The imaging process period duration was approximately 30 to 40 minutes. After technical assessment, the scoring of these acquired images was done according to the standard of image quality classification.^[15]^ Two well qualified radiologists who were well versed in HRMRI carotid image analysis evaluated the images on a scale from 1 to 5 points: images with no recognizable arterial walls and borders were assigned 1 point; images with visible walls yet unclear boundaries were assigned 2 points; images with indistinct yet recognizable boundaries were assigned 3 points; images with clear arterial walls and boundaries were assigned 4 points; and finally, images with acutely sharp boundary walls were assigned the maximum score of 5 points. All patients with unilateral carotid scores less than or equal to 2 points were excluded from the study.

### 2.4 Predictor measures

The HRMRI images with acceptable scores included in the study were in accordance with the quality classification standards. Next, the number, locations, and distributive characteristics of plaques^[16]^ were measured, and the carotid vulnerable plaques were defined as lipid rich core (type IV–V) and hemorrhage in the plaque, fibrous cap rupture, and/or calcified nodules protruding into the lumen were defined as type VI. Following this, the plaques were subsequently classified into various categories from I to VII based on the HRMRI classification criteria of the carotid artery plaque,^[17]^ wherein type I to III and type VII were considered as stable plaques, type IV–V to VI were considered as unstable plaques; at the same time, type IV to V and VI were considered as complex and vulnerable plaques. The main cause of cardiovascular events was confirmed as being in relation to the instability of the atherosclerotic plaques (vulnerability). The size of each respective plaque type was subsequently measured, including the maximum wall thickness (mm), the narrowest wall area (mm^2^), the plaque area at the narrowest wall (mm^2^), and the proportion of plaque area in the vicinity of the narrowest wall (%). In regards to the TOF sequence, the fibrous caps were presented as high signal regions. The vessel of the lumen and other compositions in the plaque was presented as low signal regions on the TOF sequence, while the T1WI, T2WI, and proton density-weighted image were all displayed respectively as either equal or high signal regions. Fibrous caps were divided into degree I of thick fibrous caps, degree II of thin fibrous caps, and degree III of ruptured fibrous caps in accordance with the preset grading standards.^[18]^ Following this the lipid composition and lipid ratio of the plaques were measured. Modified Rankin Scale (mRS) score of patients was checked.^[19]^

### 2.5 Treatment and follow-up

A total of 184 patients were treated utilizing traditional treatment approaches (neuroprotection, reducing intracranial pressure, reducing blood pressure, and antiplatelet aggregation). The remaining 148 patients were treated using modern treatments (thrombolysis, anticoagulation, and stem cell transplantation). In regards to the complete duration of the disease, all the patients were subject to follow-up methods by means of telephone calls and clinical visitations every 3 months. The mRS score^[20]^ was used for prognostic assessment. The prognosis scores were subsequently classified into categories relating to good prognosis (0–2 points) or poor prognosis (3–5 points or death). A total of 3 patients had deceased while the follow-up activities were still ongoing.

### 2.6 Statistical analysis

Data analysis was conducted using SPSS21.0 statistical software (SPSS Inc., Chicago, IL). All measurement data were presented as mean ± standard deviations (SD). Comparisons between 2 groups were conducted using paired and unpaired *t* tests. Comparisons among multiple groups were performed using the one-way analysis of variance. Enumeration data were expressed as the percentage and the comparisons between 2 groups were analyzed with chi-square tests. The prognosis analysis was conducted using multifactor logistic regression models, while a receiver-operating characteristic (ROC) curve was used to calculate the area under the curve (AUC) specificity and sensitivity areas, respectively. *P* < .05 was accepted as an indicative of statistical significance.

## 3 Results

### 3.1 The number and distribution of carotid atherosclerotic plaques in IS patients

**Figure 1:**
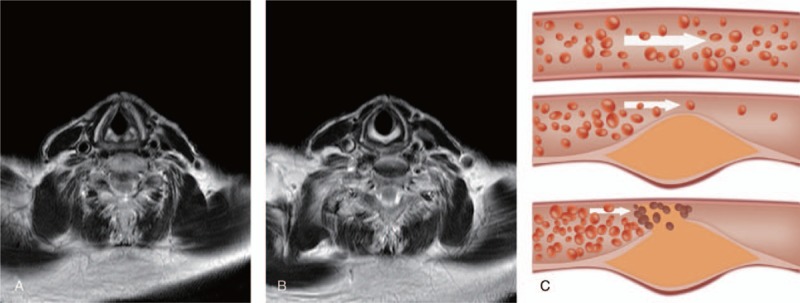
The conditions of carotid atherosclerotic plaques in ischemic stroke (IS) patients. (A) No carotid bifurcation plaque in IS patients (the matrix size was 256 × 256); (B) the carotid bifurcation plaque in IS patients (the matrix size was 256 × 256); and (C) the conditions of carotid atherosclerosis in patients with IS.

The number of carotid atherosclerotic plaques in IS patients was successfully recorded. The results revealed the total number of plaques was 551 among 338 patients. Among these patients, 6.2% had no plaque, 36.7% exhibited single plaques, while 57.1% of patients had multiple plaques. The resultant distribution of the carotid atherosclerotic plaques indicated that there was no major difference in regards to the occurrence of plaques between the left and right sides of the carotid (all *P* > .05). However, on both sides, the occurrence of plaque exhibited significant difference (all *P* < .05). In comparison to the entire carotid artery and the cervical segment of carotid artery, the occurrence of plaques at carotid bifurcation and at the site of origin of internal carotid artery was increased significantly (both *P* < .05). However, there appeared to be an insignificant difference in terms of plaque occurrence between the cervical segment of the carotid artery and the entire carotid artery as well as between the carotid bifurcation and the site of origin of the internal carotid artery (all *P* > .05). The aforementioned results are illustrated in Table 1 and Fig. 1. Figure 1A revealed evidence of plaque at the site of carotid bifurcation in patients with IS; the Fig. 1B revealed there to be no evidence of plaque in carotid bifurcation of patients with IS; finally, Fig. 1C exhibited 3 respective conditions of carotid atherosclerosis in patients with IS (without carotid atherosclerosis, with carotid atherosclerosis, and with carotid atherosclerosis plaques, respectively).

**Table 1:**
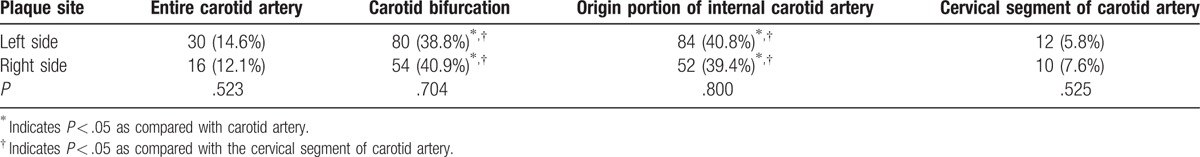
The occurrence ratio of carotid atherosclerotic plaques at different sites in schemic stroke (IS) patients (n [%]).

### 3.2 The size of different types of carotid atherosclerotic plaques in IS patients

According to HRMRI classification criteria, the carotid atherosclerotic plaques were divided into stable and unstable types. The results disclosed the values of the maximum arterial wall thickness, the arterial wall area in the narrowest point vicinity, and plaque area at the narrowest point. The proportion of plaque area at the narrowest point was significantly increased in the unstable plaque region when compared to stable plaques (all *P* < .05) (Table 2).

**Table 2:**
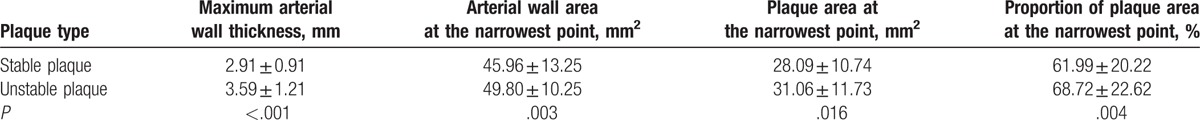
The size of different types of carotid atherosclerotic plaques in ischemic stroke (IS) patients.

### 3.3 Fibrous caps and lipid compositions of different carotid atherosclerotic plaques in IS patients

The stable plaques were mainly type I fibrous caps, while the unstable plaques were mainly type III fibrous caps. Compared with the stable plaques, the percentage of type I fibrous caps in the unstable plaques was significantly decreased, while the percentage of type III fibrous caps was significantly increased, and both the lipid compositions and lipid ratio were also increased (all *P* < .05). By contrast, there was no significant difference detected in the percentage of type II fibrous caps between the unstable and stable plaques (all *P* > .05) (Table 3).

**Table 3:**
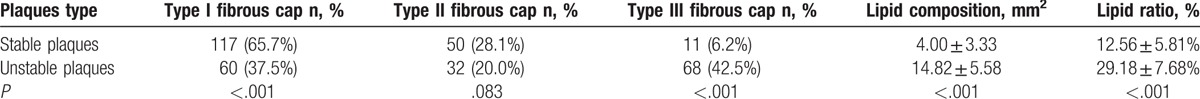
Fibrous caps and lipid compositions of different carotid atherosclerotic plaques in ischemic stroke (IS) patients.

### 3.4 Effects of different risk factors on the stability of carotid atherosclerotic plaques in IS patients

The obtained results demonstrated that gender, the history of diabetes, coronary heart diseases, obesity as well as a history of alcohol consumption and smoking in IS patients bared no major difference in the stability of carotid atherosclerotic plaques (all *P* > .05). However, in contrast age, hypertension, and hyperlipidemia in IS patients appeared to have significant adverse effects in regards to the stability of the carotid atherosclerotic plaques (all *P* < .05). It was suggested that age, hypertension, and hyperlipidemia were the main risk factors affecting the stability of carotid atherosclerotic plaques in IS patients (Table 4).

**Table 4:**
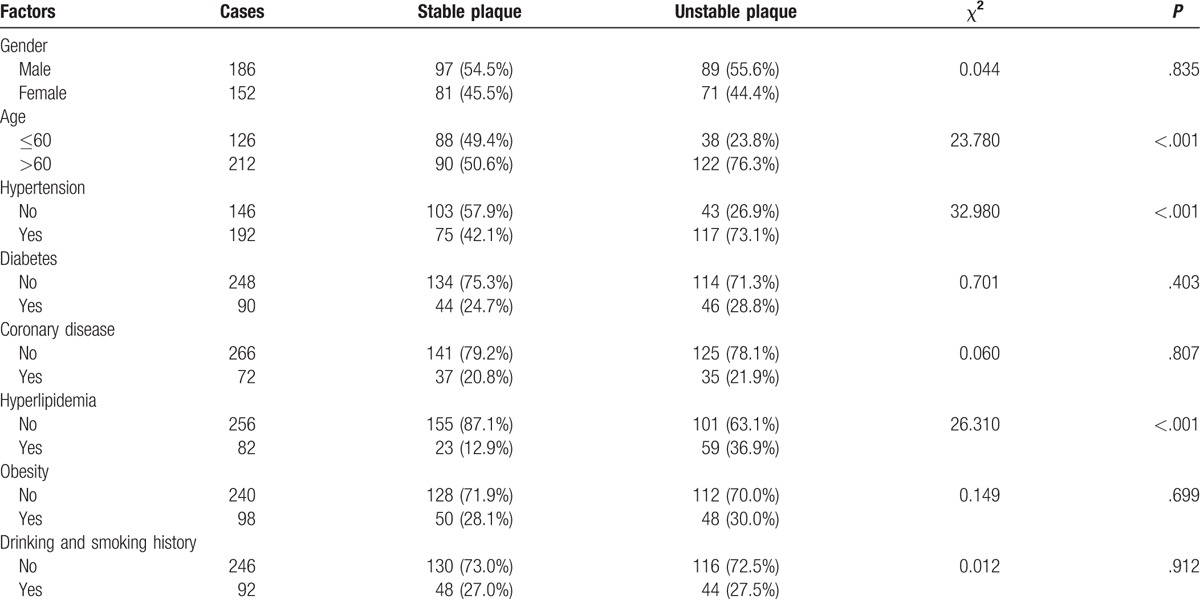
Effects of different risk factors on the stability of carotid atherosclerosis plaque in ischemic stroke (IS) patients (n [%]).

### 3.5 Univariate analysis of the factors affecting the prognosis of IS patients

During the 3-month follow-up process, 6 patients died, while 210 patients survived and displayed signs of positive prognosis, and 122 patients showed poor prognoses. The results of univariate analysis indicated that age, hypertension, hyperlipidemia, treatment regimens, fibrous cap type, plaque type, lipid composition, and lipid ratio shared significant associations in regards to poor prognoses of IS patients (all *P* < .05). The poor prognoses of IS patients were not related to gender, history of diabetes, coronary heart diseases, drinking, smoking, obesity, maximum arterial wall thickness, arterial wall area, and plaque area as well as its ratio to the narrowest point (all *P* > .05) (Table 5).

**Table 5:**
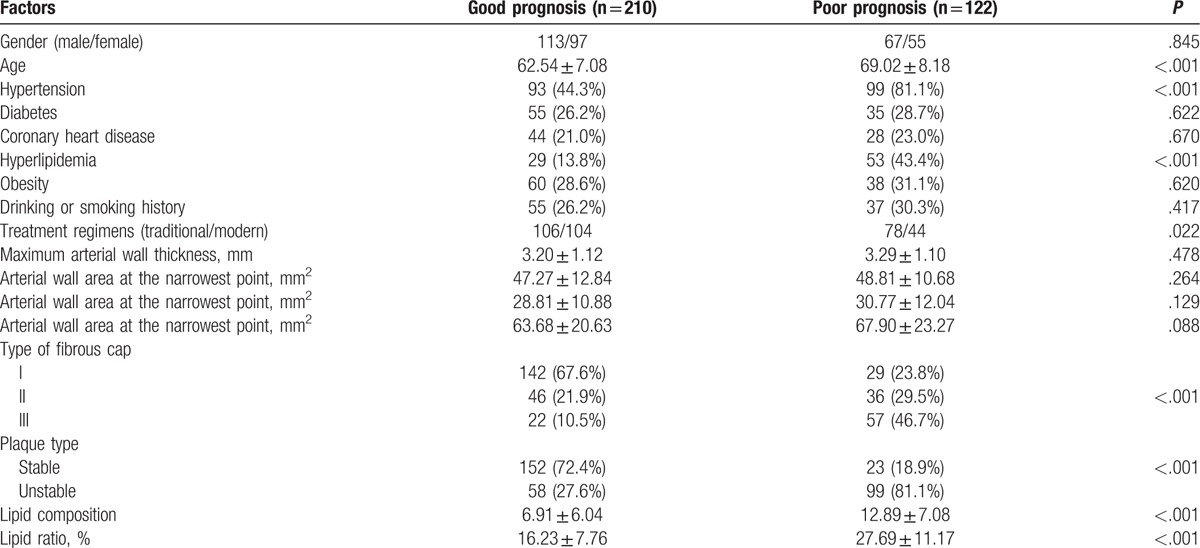
Univariate analysis of different factors affecting the prognosis of ischemic stroke (IS) patients.

### 3.6 Multivariate analysis of different factors affecting the prognosis of IS patients

Various factors associated with poor prognosis of IS patients were confirmed in the study during the univariate analysis. The aforementioned factors were further evaluated using a multivariate logistic regression model. It was subsequently established that the fibrous cap type, plaque type as well as the age of IS patients were the independent risk factors capable of influencing the prognosis of IS patients (all *P* < .05). These findings ultimately suggested that HRMRI inspections displayed promising clinical values in regards to predicting the prognosis of IS patients by determining the type of carotid atherosclerotic plaques and fibrous caps (Table 6).

**Table 6:**
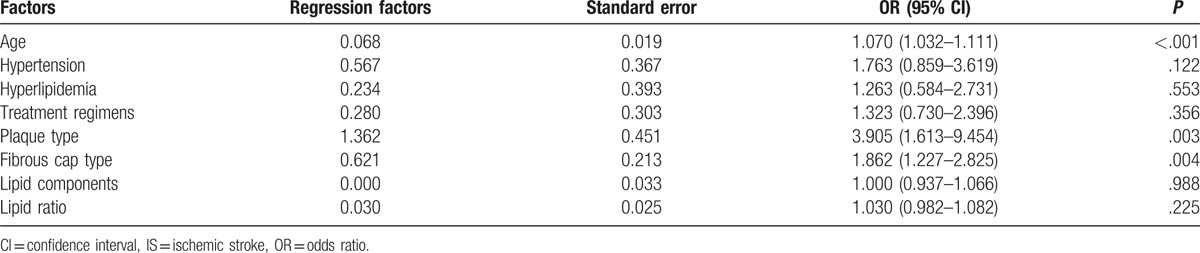
Multivariate analysis of different factors affecting the prognosis of IS patients.

### 3.7 ROC curve for determining the accuracy of IS prognosis

**Figure 2:**
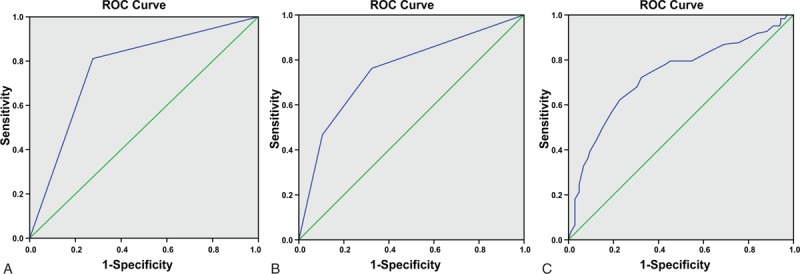
ROC curves for various risk factors in the prognosis of IS patients. (A) The ROC curve analysis for plaque types; (B) the ROC curve analysis for fibrous caps; and (C) the ROC curve analysis for age. IS = ischemic stroke, ROC = receiver-operating characteristic.

The key factors (type of plaques, fibrous caps, and age of patients) as indicators of the prognosis of IS were further analyzed using ROC curves. The AUC for plaque type was found to be 0.768 (*P* < .05), the sensitivity was 81.1% and the specificity was 72.4%. The AUC for the type of fibrous cap was 0.755 (*P* < .05), the sensitivity was 76.2% and the specificity was 67.6%. The AUC for age was 0.730 (*P* < .05), the sensitivity was 72.1% and the specificity was 67.6%. These results suggested that HRMRI had good diagnostic values in predicting the prognosis of IS patients by determining various types of carotid atherosclerotic plaque as well as fibrous caps (Fig. 2).

## 4 Discussion

IS accounts for one of the more fatal diseases currently faced worldwide, which can cause severe damage to the human body.^[21]^ As the main clinical cause of IS, carotid atherosclerosis has been indicated to be directly linked to the treatment effect, prognosis, and recurrence of IS.^[4]^ Owing to its sensitivity in measuring vascular structures,^[22]^ MRI with improved resolutions may become the epitome of medical imaging technology in the analyses of IS. Therefore, we were accordingly aided by employing HRMRI technology during our study. HRMI ultimately facilitated a comprehensive understanding of the number and distribution of carotid atherosclerotic plaques, size, lipid composition, types of plaques, and fibrous caps. This allowed for a well-timed determination of the progression of the disease, thus providing high diagnostic values for the prognosis of IS patients. Consistent with our study, Sadat et al reported that on the use of high-resolution MRI and demonstrated its ability in identifying high-risk carotid plaque and differentiate symptomatic from asymptomatic patients.^[23]^ Additionally, Gao et al^[24]^ reported that CEMRI may have an important application in the clinical risk evaluations of atherosclerosis.

This study revealed that the occurrence of plaque in IS patients to be particularly high, furthermore the plaques were found to be generally concentrated in the carotid bifurcation and at the site of origin of the internal carotid artery. This suggested that atherosclerosis generally occurs in the bifurcations and curves along blood vessels where the blood flow decreases or stops, thus resulting in a decline in the atherosclerotic plaque formation as well as a reduction in the shear stress of blood flow; in the area around the outer wall of the blood vessel where it bifurcates and at the site of origin of the internal carotid artery, the shear stress is generally minimum at the wall of the blood vessels and is prone to the formation of large plaques, eventually leading to carotid stenosis and IS.^[2]^ Atherosclerosis has shown to have a causative effect on changing the blood supply to the intracranial white spots, leading to necrosis in ischemic areas and resulting in the manifestation of IS.^[13]^ Through the systematic examination of 348 patients, it was established that 36 patients (75%) had atherosclerotic plaques, while plaque in 35 patients (94.4%) appeared to occur in the vicinity of the arch of the arteries.^[24]^ This was in consistent with the results of this study.

Our study also demonstrated that when compared to stable carotid atherosclerotic plaque, the vascular walls of the unstable plaques were thicker. Furthermore, the wall areas at the narrowest points were larger, the plaque areas and ratios at the narrowest points were greater, the lipid composition ratios were higher, and the fibrous caps were mainly of the type III variety. Plaque fibrous caps displayed a stabilizing effect, while their lipid cores were among the destabilizing factors.^[25]^ Additionally, other factors affecting the stability of the plaque included the presence of inflammatory cells as well as the stress changes around the plaque.^[26,27]^ The composition of unstable plaque makes them prone to detachment and can cause distal embolization. These unstable structures also tend to cause the rapid progression of vascular blockages manifested by thrombosis and plaque hemorrhage.^[27]^ By examining 94 cases of abnormal plaque occurrence among 110 patients indicated that the incidence of IS in patients with unstable plaque was significantly higher than patients with stable plaque occurrence.^[28]^

This study also highlighted age, hypertension, and hyperlipidemia as the risk factors affecting the stability of the carotid atherosclerotic plaque in IS patients. It has been reported that the age of a patient was an uncontrollable factor of IS and the exposure to different risk factors increases at an older age including an increase of the load at the arterial linings, leading to endothelial damages and affecting the stability of carotid atherosclerotic plaques in IS.^[29]^ Higher blood pressure was found to be associated with an increased risk of death or disability in patients with symptomatic intracranial artery stenosis.^[29]^ In hyperlipidemia patients, an increase in cholesterol level promotes platelet aggregation and increases plasma viscosity, thus increasing vascular resistance and slowing the blood flow, which may cause the formation of blood clots and increase the risk of IS.^[5]^ Kim et al^[30]^ examined 83 IS patients and indicated particular risk factors including patient age and high blood pressure as being closely related to the manifestation of more severe atherosclerotic plaque occurrence.

However, there were certain limitations faced during this study. First, we placed a strong emphasis on HRMRI and it was consequently insufficient in attesting the carotid atherosclerotic plaques. Second, it is well known that the hyperlipidemia is an important risk factor affecting the stability of carotid atherosclerotic plaques; however, the effect of hyperlipidemia on HRMRI in the diagnosis of cervix AS plaques was not evaluated during this study.

In conclusion, HRMRI exhibited a particularly high diagnostic value in the prognosis of IS patients by detecting the type of carotid atherosclerotic plaque as well as the fibrous caps. However, because of the limitations exhibited in our study, the effects of hyperlipidemia on the stability of carotid atherosclerotic plaques need to be clinically evaluated for the further validation of the findings of this study.

## Acknowledgments

The authors thank all the people for assistance in editing this manuscript.
